# Epidemiology of Suicide in Western Odisha During COVID Pandemic: A Cross-Sectional Analysis

**DOI:** 10.7759/cureus.21438

**Published:** 2022-01-19

**Authors:** Sanjeeb K Mishra, Ashok K Panigrahi, Smita K Panda, Satya Sai Panda, Gitarani Choubey, Shwetlana Panda, Sushree Behera, Subrat K Pradhan

**Affiliations:** 1 Department of Epidemiology, Indian Council of Medical Research, Chennai, IND; 2 Community Medicine, Veer Surendra Sai Institute of Medical Sciences and Research, Sambalpur, IND; 3 Pharmacology, Veer Surendra Sai Institute of Medical Sciences and Research, Sambalpur, IND; 4 Forensic Medicine, Veer Surendra Sai Institute of Medical Sciences and Research, Sambalpur, IND

**Keywords:** covid-19 related mental issues, odisha, epidemiology, india, burn, hanging, poisoning, lockdown, covid pandemic, suicide

## Abstract

Introduction

Suicide is the act of deliberately killing oneself. It is a leading cause of mortality worldwide. Each year, more than seven lakh people end their lives globally. India is the worst-affected country in Southeast Asia. Both the genders and all age groups are affected. The COVID pandemic has led to the disruption of routine life and business. The proportion of deaths due to suicide was 9.4% among all deaths reported for autopsies by a study in the same mortuary over a seven-year period. Increased stress and anxiety have been postulated to lead to suicide. Our study objective is to describe the epidemiology of suicide during the early COVID pandemic (lockdown period).

Methods

This is a record-based cross-sectional study. We have analyzed the post-mortem reports for six months starting from April 1, 2020. Descriptive analysis was performed with Epi Info version 7 (Centers for Disease Control and Prevention, CDC, Atlanta, GA, USA).

Results

During the study period, 340 cases were classified as deaths due to suicide, out of a total of 891 mortalities. The median age for females was 26.5 and for males, it was 30. The male to female ratio was 1.8:1. Most of the deceased (39.8%) were in their third decade, followed by the fourth decade (18.9%), second decade (15%), and fifth decade (12.98%), respectively. Poisoning was the leading method used for suicide, accounting for 238 (70.1%) deaths, followed by hanging (11.8%), burns (6.8%), jumping from a height (6.5%), and jumping in front of the train (4.13%). Self-immolation (burning) was a common mode of suicide for females. Most of the suicides (71.4%) took place from the evening to midnight.

Conclusion

There was a fourfold rise in suicides during the period compared to previous data. Productive age groups are affected more. A large-scale multi-centric study in community settings for estimation of the true burden is the need of the hour. A multi-sectorial public health approach is needed to prevent untimely death due to suicide.

## Introduction

Suicide is one of the leading public health problems globally, and every year, more than seven lakh people die due to suicide [1], and India contributes more than one-fourth of the global burden of suicide [2]. Though it is a global phenomenon, over 77% of suicides occurred in lower-middle-income countries (LMICs) in the year 2019, and in the same year, it was the fourth leading cause of death among those aged 15-29 years old [1]. According to an estimate by the World Health Organization (WHO), India has the highest suicide rates in Southeast Asia and ranks third in female suicide rates in the entire world. It is the leading cause of death among people in their third and fourth decades, only behind road traffic accidents [3]. In India, the overall suicide rate varies from 10.3 to 16.3 per lakh population [3,4]. It amounts to one life lost every three seconds by suicide. Different studies from India have found poisoning as the most common mode of suicide, followed by hanging [3,5-8]. However, the National Crime Records Bureau of India (NCRB) reported hanging as the most frequent mode of committing suicide [4]. Studies conducted during the lockdown period have reported higher suicide rates in different countries, including India [9]. Many people were stranded, lost their jobs due to the lockdown, which was reported to cause stress and suicidal behavior among people of low socio-economic class [10]. In most cases, there is a preventable cause of suicide that can be identified and rectified with research and early intervention [3,7,8]. According to the WHO, only 38 countries reported having a national suicide prevention strategy [1]. The suicide mortality rate is an indicator of target 3.4 of the Sustainable Development Goals by 2030 to reduce by one-third premature mortality from non-communicable diseases [11]. Information on the most commonly practiced suicide methods is important to devise prevention strategies. With this background, we conducted the study with the objective of describing the epidemiology of suicide in western Odisha during the lockdown period of the COVID pandemic.

## Materials and methods

We conducted a cross-sectional study at the Veer Surendra Sai Institute of Medical Sciences and Research, a tertiary care institution catering to western Odisha. The study included all the deaths presented for post-mortem and notified as suicide from April 1 to September 30, 2020. In India, a lockdown was imposed on March 24 and extended till May 31 in four phases. A partial lockdown imposed till September 30, 2020. Only 340 were classified as suicide, out of 891 cases examined during the study period.

Criteria for inclusion and exclusion of cases

(1) All the cases that had an allegation of suicide in inquest report and corroborating autopsy findings and circumstantial evidence. (2) Inquest report without allegation but with circumstantial evidence and post-mortem findings in line with suicide. (3) All victims of accidents, homicide, and unclassified deaths were excluded.

Materials

(1) Post-mortem examination reports. (2) Inquest report. (3) Documents received from investigating officers. (4) Hospital records in cases that were admitted to a hospital before death. (5) Interview with the relatives.

Variables

Data were recorded in a pre-designed format to cover the classical triad of distribution in epidemiology, i.e., time, place, and person. Time variables include event time, time to reach medical services, and time of death. Place variables include residence (rural and urban), place of incident, and place of treatment. Person variables include age, sex, education qualification, history of coexisting illness, and addiction. The mode or manner of committing suicide was the outcome variable. The information collected was analyzed using appropriate statistical tools.

Statistics

Data were entered into Microsoft Excel (Microsoft Corporation, Redmond, Washington, USA) and checked for completeness. Data were analyzed using Epi Info version 7 (Centers for Disease Control and Prevention, CDC, Atlanta, Georgia, USA) to describe the frequency, mean, median, confidence interval, and range. For graphical representations, GraphPad Prism Version 8.02 (Released February 6, 2019, GraphPad Software Inc., San Diego, California) was used.

## Results

A total of 891 deaths were reported at the mortuary during the study period, out of which 340 deaths were due to suicide (38.2%). One suicide case was unclaimed and was excluded from the analysis due to missing information. The characteristics of the suicide cases are displayed in Table 1.

**Table 1:** Characteristics of the suicide cases (N =339).

	Number	Poisoning	Hanging	Burn	Jump from height	Jump in front of train	Drowning
Total	339	238	40	23	22	14	2
Gender	Male (219)	161	23	3	20	10	2
	Female (120)	77	17	20	2	4	0
Age	14-30 year (186)	119	31	20	11	5	0
	31-50 year (108)	77	8	3	11	7	2
	51 and above (45)	42	1	0	0	2	0
Education	Nil (1)	1	0	0	0	0	0
	Primary (9)	4	2	1	2	0	0
	Secondary (40)	11	4	1	14	8	2
	High-school (143)	105	18	8	6	6	0
	Graduate and above (146)	117	16	13	0	0	0
Marital status	Married (226)	161	20	19	15	9	2
	Unmarried (110)	74	20	4	7	5	0
	Widow (3)	3	0	0	0	0	0
Place of event	Home (242)	193	25	17	7	0	0
	Work place (22)	8	4	6	4	0	0
	Outside (75)	37	11	0	11	14	2
Time of event	6 am-12 pm (25)	1	6	10	7	0	1
	12 pm-6 pm (62)	21	16	11	13	0	1
	6 pm-12 am (242)	216	16	2	2	6	0
	12 am-6 am (10)	0	2	0	0	8	0
Time to reach hospital	0-2 h (20)	15	2	2	1	0	0
	2-6 h (252)	194	21	19	17	0	1
	6-24 h (31)	28	0	2	1	0	0
	Received dead (36)	1	17	0	3	14	1

Age ranged from 14 to 85 with a median age of 30 and interquartile range (IQR) = 23, 43. Most deceased (39.8%) were in their third decade, followed by the fourth decade (18.9%), the second decade (15%), and the fifth decade (12.98%), respectively. Out of all the cases, 220 (64.6%) were males, and 119 (35.4%) were females, with a male to female (M:F) ratio of 1.8:1. The highest M/F ratio was in the age group of 61-70 at 4.33 and the lowest was for a person aged 11-20 at 0.89. The frequency of males was higher in all age groups except for the second decade. The median age for females was 26.5 (IQR 21, 37.5) and for males was 30 (IQR 25, 45).

The majority of the cases belonged to the Sambalpur (42.43%) district, followed by Bargarh (29.95%), Sundergarh (12.69%), and Jharsuguda (9.28%). Most deaths were from rural areas (91.1%). Of the deceased, 226 (66.6%) were married, 110 (32.45%) were unmarried, and three were widows. As per educational status, most were either graduates or higher (43.07%), followed by higher secondary (42.18%). The majority (80.2%) did not have any addiction, 59 were regular tobacco users, three were alcoholics, and five used both. Most deceased (85%) did not have any diagnosed comorbidity. Hypertension (6.1%), psychiatric illness (4.1%), audio-visual issues (12, 4.0%), and diabetes (9, 2.6%) were the leading comorbidities.

The manner for committing suicide 

**Figure 1:**
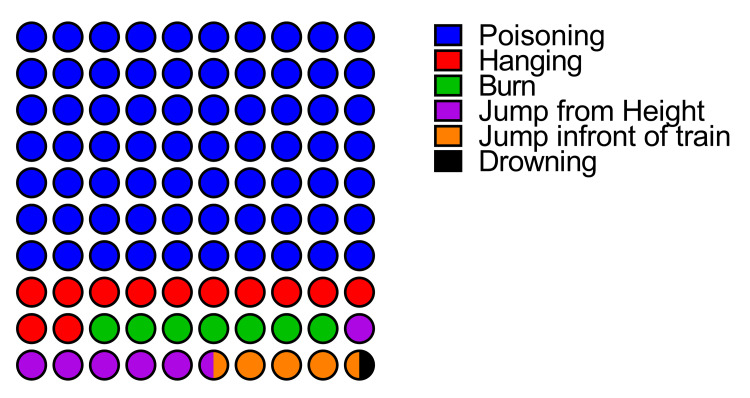
Dot plot showing distribution of mode for committing suicide.

Poisoning was the leading method used for suicide, accounting for 238 (70.1%) deaths, followed by hanging (11.8%), burn (6.8%), jumping from a height (6.5%), and jumping in front of the train (4.13%) (Figure 1).

**Figure 2:**
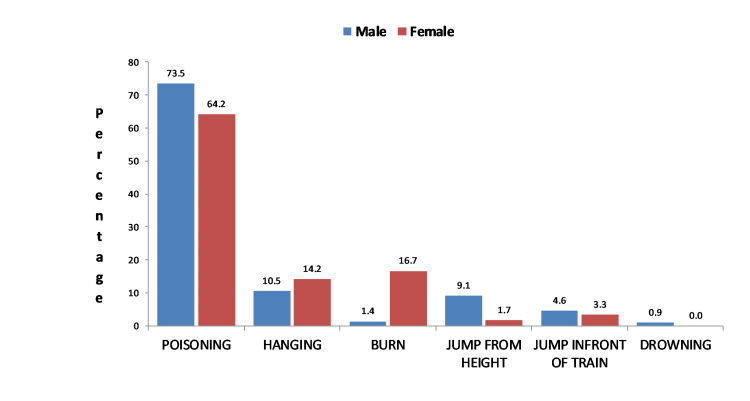
Gender-wise distribution of modes for committing suicide.

Poisoning was the leading mode among all age groups in both sexes. In males, hanging (10.5%) and jumping from height (9.13%) were the second and third leading manner in comparison to burns (16.67%) and hanging (14.17%) in females (Figure 2).

Most (86.96%) burn cases occurred in the third decade of life. Hanging was more than three times more common in urban areas (34.48%) than in rural areas (9.71%) among manners adopted for suicide. Unmarried cases used hanging twice more commonly than married (18.18% vs. 8.85%), conversely married deceased adopted burn two times more frequently than unmarried cases (8.41% vs. 3.64%). 242 (71.5%) of the suicides took place in the home, 22(6.5%) in the workplace, and rest 75 (22.1%) outside.

**Figure 3:**
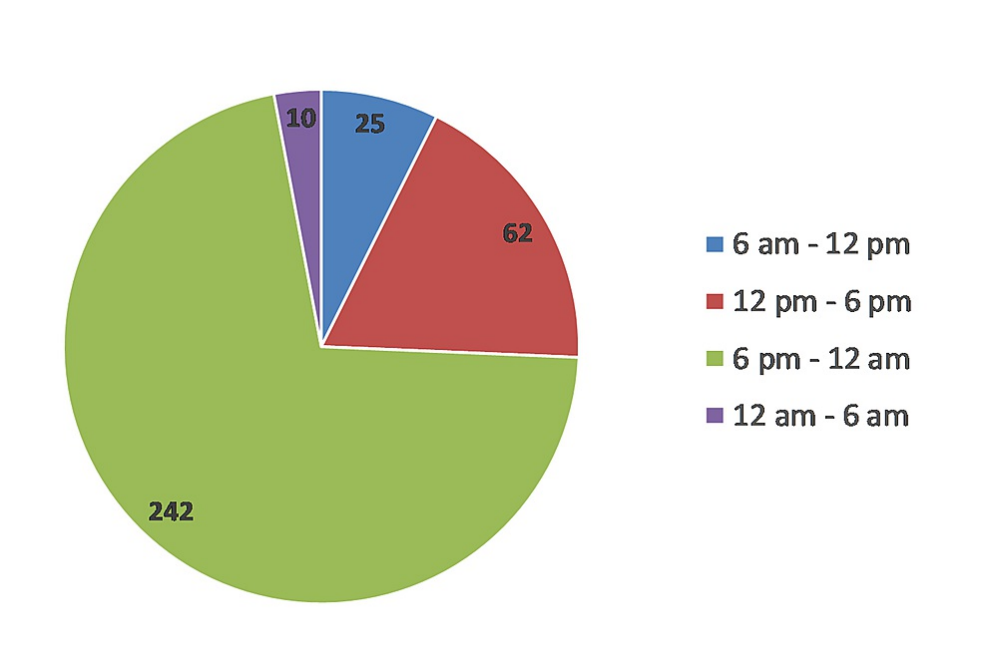
Time distribution of suicide cases.

Time of event-242 cases occurred between 6 pm and 12 am and 62 cases between 12 pm and 6 pm, which constitutes 89.7% of the cases (Figure 3). Thirty-six cases died before reaching the treatment center; 17 were hanging, 14 were jumping in front of the train, three were jumping from a height, and one was poisoned. Only 20 (5.9%) cases reached the treatment center within two hours; 15 were poisoning cases.

## Discussion

During the study period, 891 cases were reported to the mortuary, of which 340 were suicides (of which one case was excluded due to incomplete information). The proportion of deaths due to suicide was 38.2% among all death reported for autopsies. This is considerably higher than the 9.4% reported by a study in the same mortuary over a seven-year period [12]. This increase is in line with the postulated theories of increased stress during lockdown due to fear of the disease, contracting COVID, financial hardship, and discrimination [13,14]. Published literature has shown a twofold rise in suicide cases in Punjab, Himachal Pradesh, and West Bengal during the lockdown [10], and our study reported a fourfold rise. Studies have highlighted the increased suicidal ideation during epidemics due to various factors, which also explain these high suicide rates [15]. This may also be explained because the largest share of autopsy cases are attributed to road traffic accidents, which were substantially lower in the lockdown period due to restricted vehicle movement and recorded more than a 12% reduction from the previous year [4]. Analysis of suicide trends in high-income countries did not show any increase in the earlier recorded rates. However, the economic, social, and healthcare scenarios differ from those of a country like India [16].

Our study reported poisoning (70.1%) as the most common mode of suicide, followed by hanging (11.8%), which is similar to previous studies conducted in India [3,5-7,17]. Our results contrast with the recent NCRB report, which states that hanging is the leading mode (57.8%). This discrepancy has been highlighted by previous studies and the fact that the NCRB report is based only on the first information report (FIR). Until recently, suicide was criminalized and stigmatized in India, leading to underreporting [4-6]. Another possible explanation for the high poisoning rate is the rural predominance of autopsy cases (91%), where access to pesticides like organophosphates is relatively easy [5]. A study conducted during the pandemic also reported hanging as the leading model for committing suicide. However, it was based on a media report that tended to highlight the registered urban cases in the NCRB database [14]. Self-immolation (burn) was found to be the second most adopted mode for committing suicide among females (16.67%), and similar conclusions were made in previous studies [5,6,8,17].

More than half of the cases were younger than 30 years, with the highest prevalence in the third decade at 39.8%, similar to most published studies from India [2,3,8]. The females were younger than the deceased males (median 26.5 vs. 30), similar to other studies [5,6,8]. Overall, the male to female ratio among the deceased was 1.8, and this ratio showed progression as age advanced. A similar ratio was reported by a study based on NCRB reports [5]. The M:F ratio was less than one in persons in their second decade, indicating a higher prevalence of female suicide in the younger population; other studies also report similar findings [4-6,8]. The majority of the deceased education status was either higher secondary (42.2%) or graduate and above (43%), which is substantially higher than the NCRB report (higher secondary; 15.9%, graduate and above; 4%) [4]. The majority (85%) did not have any diagnosed comorbidity, and this finding is similar to the study by Panigrahi et al. that reported 89% without any comorbidity. Psychiatric illness was present in 4.1% of the deceased and is similar to the 4.5% reported by Panigrahi et al. [14].

Most studies in India have depended upon the NCRB data and concluded that there is gross underreporting in both suicide and attempted suicide owing to various factors, among which section 309 of the Indian penal code is the leading hurdle that classifies suicide attempt as a punishable offense [2,5,6]. In this context, a large-scale study to estimate the national burden of suicide and attempted suicide and identify risk factors needs to be carried out. A public health approach is required for the development and implementation of national suicide prevention strategies.

## Conclusions

Our study describes the epidemiological characteristics of suicide cases during the lockdown period of the COVID pandemic. There was a fourfold rise in suicides during this period as compared to previous data from the same mortuary. Three-fourths of cases were younger than 40 years old, emphasizing the need to intervene effectively to prevent these untimely deaths. Restricting access to means of suicide, interaction with the media for responsible reporting, training young people in their life skills, and early identification, management, and follow-up measures need to be adopted on a large scale.
